# Memory, Future Thinking, and the Self. In Honour of Martial Van Der Linden

**DOI:** 10.5334/pb.1074

**Published:** 2021-09-15

**Authors:** Arnaud D’Argembeau

**Affiliations:** 1 University of Liège, BE

**Keywords:** autobiographical memory, future thinking, self, goals, neuroimaging

## Abstract

Over the past 20 years, much progress has been made in understanding the relations between memory and future thinking, and their role in shaping our sense of self and identity. My own interest in these research questions owes much to Martial Van der Linden, with whom I had the chance to interact closely for several years. The purpose of this article is to pay tribute to him by reviewing the work we initiated together on autobiographical memory and future-oriented thinking. I first review our early work on the relationship between memory and future thinking and discuss their role in self-representation. Then, I provide an overview of the underlying neural bases and describe the alterations of autobiographical thinking that characterize certain psychological disorders. Finally, I outline an integrative framework that I recently proposed to account for the cognitive structure of past and future autobiographical thinking.

Our sense of self and identity is based on the construction of a life story that integrates and gives meaning to our experiences (McAdams, 2001). This personal narrative is particularly inhabited by the people who have played a prominent role in our lives. For me, Martial Van der Linden is certainly one of these important figures who shaped part of my identity and determined my professional life. The purpose of this article is to pay tribute to him by reviewing the work we initiated together on the relations between autobiographical memory and future thinking, as well as their roles in the construction of the self. First, I present our early work on the relations between memory and future thinking and describe how it contributed to the nascent field of research on episodic future thinking. Then, I discuss the roles of autobiographical memories and future thoughts in self-representation, provide an overview of their neural bases, and describe their alterations in some psychological disorders. Finally, I briefly sketch an integrative framework that I recently proposed to account for the structure of past and future autobiographical thinking. Please note that my aim here is not to provide a comprehensive review of the role of memory and future thinking in the sense of self (see e.g., Conway et al., 2019; Fivush, 2011; Klein & Gangi, 2010; McAdams, 2013; Prebble et al., 2013), but rather to give a glimpse of the path that Martial and I have travelled together in exploring this issue.

## Memory and future thinking are closely linked

While memory obviously refers to the past, from an evolutionary perspective, its fundamental function is to aid the organism in anticipating and planning for the future (Klein, 2013; Suddendorf & Corballis, 2007; Tulving, 2005). Surprisingly, however, it is only quite recently that empirical research on future-oriented thinking has taken off. Tulving (1985) was one of the first to note the importance of episodic memory for imagining the future, based on his observations of an amnesic patient. In a seminal paper drawing on Tulving’s work, Atance and O’Neill (2001) defined *episodic future thinking* as the “ability to project the self forward in time to pre-experience an event” (p. 537). At the time, I was investigating the influence of emotion on the phenomenological characteristics of autobiographical memories as part of my PhD thesis under the supervision of Martial (D’Argembeau et al., 2003), and when I first read the Atance and O’Neill’s paper, I immediately recognized the potential interest of testing the relations between memories and future thoughts using our measures of phenomenological characteristics. Martial was enthusiastic about this idea and thus we decided to run a study in which we assessed whether mental representations of past and future events were similarly influenced by event characteristics; our rationale was that if remembering and future thinking are indeed related, they should be similarly influenced by features of the represented events. To test this hypothesis, we manipulated the emotional valence and temporal distance of events by asking participants to remember positive and negative events that they experienced in the recent or distant past (i.e., last year vs. the past 5–10 years) and to imagine positive and negative events that might happen to them in the near or far future (next year vs. 5–10 years from now). We found that, although future thoughts were generally less detailed than memories, the two types of representations were influenced by the event characteristics in the same way, with positive and temporally close events containing more sensory and contextual details than negative and temporally distant events (D’Argembeau & Van der Linden, 2004).

In a subsequent study, we sought to gather additional evidence for the relationship between remembering and future thinking by examining whether individual differences in dimensions that affect memory for past events similarly influence the experience of projecting oneself into the future. We notably found that people who possess a higher capacity for visual imagery experience more visual and other sensory details both when remembering past events and when imagining future events (D’Argembeau & Van der Linden, 2006). Together, the results of these two studies laid the ground for neuroimaging research on episodic future thinking (Addis et al., 2007; Szpunar et al., 2007) and contributed to theoretical advances in understanding the constructive nature of memory and its functions, most notably the idea that episodic memory allows us to utilize details from past experiences to imagine events that have never been experienced as such in the past—the constructive episodic simulation hypothesis (Schacter & Addis, 2007).

Beyond merely showing that remembering the past and imagining the future are closely linked, we sought to better understand underlying processes. To do so, we examined the extent to which various cognitive processes and abilities (i.e., executive control, visuospatial processing, relational memory, self-consciousness, and time perspective) were predictive of the accessibility, specificity, and level of detail of mental representations of past and future events. We found that executive processes, in particular the ability to organize and monitor memory retrieval, played a general role in accessing and constructing mental representations of past and future events at different levels of abstraction, whereas visuospatial processing and self-consciousness contributed more specifically to the production of sensory details when imagining events (D’Argembeau, Ortoleva, et al., 2010). These results suggest that future thinking involves a collection of component processes that support different facets of future event representations.

The subjective experience of projecting oneself into the future is indeed a complex, multi-dimensional phenomenon that not only involves the construction of a mental scene (as is the case for any imagined event) but also a sense of “pre-experiencing” the future (Atance & O’Neill, 2001; Tulving, 1985). Understanding the determinants of these phenomenological qualities is important because they shape our beliefs about what might lie ahead (Ernst & D’Argembeau, 2017), and in turn influence our decisions and actions (Libby et al., 2007). To shed light on this issue, Martial and I investigated the specific contribution of various event features to different aspects of the phenomenology of episodic future thoughts (D’Argembeau & Van der Linden, 2012). We found that the vividness of an episodic future thought was largely dependent on the familiarity of its constitutive elements (i.e., the envisioned location, persons and objects). More importantly, the subjective sense of pre-experiencing the future was not only based on the sensory–perceptual qualities of the mental representation but was also modulated by the personal importance of the imagined event. This latter finding led us to suggest that the essence of episodic future thinking—the sensation of mentally visiting one’s personal future—lies, in part, in the connection of imagined events with personal goals, an idea that is developed in the next section.

## The roles of past and future thoughts in self-representation

An important function of autobiographical memory is to support our sense of self and identity (Conway et al., 2004; Fivush, 2011; Habermas & Bluck, 2000; McAdams, 2001; Prebble et al., 2013). As object of knowledge, the self relies on a multi-layered representational structure that includes abstract representations of our personal characteristics such as our traits (e.g., “I am timid”), general knowledge about the periods and events that constitute our life (e.g., “when I was in primary school, we used to visit my grandma on Sundays”), and memories of specific happenings in our past (e.g., “the time I broke my leg in grandma’s garden”) (Conway, 2005). These different forms of self-knowledge are functionally isolable; for example, amnesic patients are able to make trait self-descriptiveness judgements despite their inability to recollect personal events relevant to those judgements (Klein & Lax, 2010). Under normal circumstances, however, the different layers of self-representation interact closely with each other, such that memories of past experiences sustain and exemplify our images of ourselves (Charlesworth et al., 2016; Grilli, 2017; Rathbone et al., 2008). In particular, a special class of memories—referred to as *self-defining memories*—serve as the building blocks of narrative identity—the stories we construct about the self to develop and sustain a sense of personal unity and purpose (McAdams, 2001; Singer et al., 2013). These are vivid, affectively intense, and often-rehearsed memories that involve important themes and concerns in our life (e.g., achievement, intimacy) (Singer & Moffitt, 1991). The process of linking these recollected experiences to the conceptual structures of the self is key to the construction of life narratives and is generally predictive of psychological health and well-being (Habermas & Bluck, 2000; Philippe et al., 2012; Singer et al., 2013; Thorne et al., 2004).

Our self-image is of course not limited to the past but also includes representations of what we might become, would like to become, and are afraid of becoming in the future (Markus & Nurius, 1986). The sense of self and identity can thus be fostered by the imagination of meaningful events that we anticipate to happen in the future; indeed, several studies have shown that future events are, on average, considered as more important to the self than past events (e.g., Addis et al., 2008; Berntsen & Bohn, 2010; D’Argembeau & Van der Linden, 2006). To shed light on this future aspect of the self-image, Martial and I introduced the notion of *self-defining future projection* as the future counterpart of self-defining memories: a subcategory of imagined future events that relate to important personal goals and provide key information for self-understanding (D’Argembeau, Lardi, et al., 2012). In the same way that self-defining memories support conceptual knowledge about present and past selves, self-defining future projections ground and exemplify our conceptions of ourselves in the future. For example, a teenager who envisions her future self as a mother may nourish this self-image with the imagination of future scenarios that incarnate this possible self (e.g., imagining herself at the maternity hospital, taking the children to school, playing with them in the backyard, and so on). In two studies, we found that people can readily produce these types of self-defining future events and that individual differences in the characteristics of self-defining narratives manifest themselves in similar ways for past and future events (D’Argembeau, Lardi, et al., 2012). Furthermore, as with self-defining memories, self-defining future projections not only represent the concrete content of future events but also sometimes their broader meaning and potential implications for the self. Continuing this line of research, Demblon and D’Argembeau (2017) showed that self-defining memories and future thoughts are organized in networks of events according to their themes and identity motives (e.g., efficacy, belongingness, self-esteem, and so on).

At the heart of future-oriented self-representations lie the articulation of and investment in *personal goals* (McAdams, 2013). Generally speaking, goals are cognitive representations of desired states or outcomes (Austin & Vancouver, 1996), and personal goals can be defined as personally salient objectives that people pursue in their lives (Emmons, 1986; Little, 1983; Milyavskaya & Werner, 2018). Goal-related knowledge is represented at different levels of abstraction, from very general aspirations (e.g., being a successful person) to concrete and specific objectives (e.g., striving to arrive at a particular meeting on time); these different goal representations are organized in a hierarchy in which higher-order goals determine the content of lower-order goals (Austin & Vancouver, 1996). Thus, an important function of episodic future thinking is to provide a detailed, quasi-experiential representation—a simulation—of what it would be like to be in a desired end state, and to mentally try out various steps and consider potential obstacles to reaching that state (D’Argembeau, 2016). According to this view, personal goals drive and constrain future event representations, which in turn motivate and guide goal pursuit. And indeed, research has shown that personal goals play a prominent role in the construction and organization of episodic future thoughts (Ben Malek et al., 2018; D’Argembeau & Demblon, 2012; D’Argembeau & Mathy, 2011; Lehner & D’Argembeau, 2016).

Another important dimension of the self-image is its emotional valence. Most people hold flattering views of themselves and process information in ways that maintain or increase the positivity of their self-image (Leary, 2007; Sedikides & Gregg, 2008). One way in which this positivity bias manifests itself is in how people envision their personal future (Taylor & Brown, 1988). Our work has indeed shown that positive future thoughts are represented more vividly than negative future thoughts (D’Argembeau & Van der Linden, 2004; see also Rasmussen & Berntsen, 2013) and are experienced more frequently in daily life (Barsics et al., 2016; D’Argembeau et al., 2011). This positivity bias appears to be particularly pronounced for self-defining events and may help support self-esteem (D’Argembeau, Lardi, et al., 2012; D’Argembeau & Van der Linden, 2008) and stimulate goal-directed behaviour (D’Argembeau & Van der Linden, 2007).

## The neural correlates of autobiographical thinking

Martial played an important role in the introduction of neuroimaging techniques at the University of Liège in the 90s (see e.g. Salmon et al., 1996). A the end of his career, however, he was somewhat critical of neuroimaging studies and was convinced of their usefulness only insofar as they informed and constrained cognitive theories (for further discussion, see Henson, 2005; Mather et al., 2013). In my view, research on autobiographical memory and self-referential processing is one area in which functional brain imaging contributed significantly to understanding the cognitive architecture underlying self-representation. For example, the finding that imagining the future depends on much of the same neural machinery that is needed for remembering the past played a key role in the development of novel theoretical perspectives on the functions of memory (Schacter et al., 2007).

Neuroimaging studies have also played a role in dissecting different forms of self-representation and identifying their underlying commonalities and differences. For example, representations of personal traits, knowledge of personal facts, and memories of specific events are associated with unique brain activations, showing that different types of self-representation are dissociable (Martinelli et al., 2013). Importantly, however, the brain regions that have been associated with different types of self-representation—including the medial prefrontal cortex, medial and lateral temporal areas, the posterior cingulate cortex, and inferior parietal lobes (Kim, 2012; Renoult et al., 2012; Svoboda et al., 2006)—are highly interconnected and form a coherent network (which largely corresponds to the “default network”; Buckner et al., 2008), thereby supporting the view that distinct self-knowledge domains interact closely with each other (Conway, 2005).

In our own work, we used neuroimaging to test the hypothesis that autobiographical reasoning—a process of reflective thinking through which we form links between disparate elements of our life and the self (Habermas & Bluck, 2000)—is more than the mere remembering of past events (D’Argembeau et al., 2014). During fMRI scanning, participants were asked to approach a set of self-defining memories in two different ways: in some trials, they remembered the concrete content of the events, whereas in other trials they reflected on the broader meaning and implications of their memories. We found that a number of brain regions within the autobiographical memory network showed differential activity depending on the way people approached the same self-defining memories. Specifically, a left-lateralized network composed of the dorsomedial prefrontal cortex, inferior frontal gyrus, middle temporal gyrus and angular gyrus was more active when participants reflected on the personal significance and implications of their memories (i.e., autobiographical reasoning), whereas other brain regions commonly engaged in autobiographical memory retrieval (i.e., the posterior cingulate/retrosplenial cortex, precuneus, amygdala/hippocampus, parahippocampal gyrus, dorsolateral prefrontal cortex and medial orbitofrontal cortex) showed higher activation when participants focused on the concrete content of the events and attempted to re-experience them in their original context. These findings support the notion that autobiographical reasoning and the construction of personal narratives go beyond mere remembering in that they require deriving meaning and value from past experiences.

The role of personal goals in episodic future thinking discussed above is also supported by neuroimaging evidence. If goal processing is an integral part of episodic future thinking, it should be associated with specific regions within the brain network that is activated when imagining future events (Benoit & Schacter, 2015). To test this hypothesis, we asked people to imagine future events that were related to their personal goals, as well as future events that were plausible but unrelated to their goals, and found that the medial prefrontal cortex and other midline regions were more activated when imagining goal-related future events (D’Argembeau, Stawarczyk, et al., 2010). Furthermore, meta-analyses of neuroimaging studies of episodic future thinking, on the one hand, and of personal goal processing, on the other hand, showed that the two sets of studies were associated with overlapping activation in several brain regions and most notably in the medial prefrontal cortex (Stawarczyk & D’Argembeau, 2015). Taken together, these findings suggest that the medial prefrontal cortex may contribute to the processing of personal goals when imagining future events.

Although the medial prefrontal cortex is consistently involved in episodic future thinking, and more generally in self-referential thinking, its exact function remains unclear. One possibility is that it plays a role in appraising or representing the subjective value or significance of self-related information (D’Argembeau, 2013). This account is supported, for example, by the finding that medial prefrontal activity increases linearly with the personal importance of self-referential contents (Andrews-Hanna et al., 2010; D’Argembeau, Jedidi, et al., 2012). Another, not necessarily mutually exclusive, possibility is that the medial prefrontal cortex mediates the integration of multiple representational levels during self-referential thought (see e.g. Brod et al., 2013, for evidence that the medial prefrontal cortex is involved in the integration of information to pre-existing knowledge structures). For example, when people engage in episodic future thinking, the medial prefrontal cortex may integrate imagined events with higher-order autobiographical knowledge, and may organize specific events in broader themes and causal sequences. Supporting this view, we found that the processing of event clusters (i.e., events that are part of higher-order themes and sequences) is associated with increased activation in the medial prefrontal cortex and with greater functional connectivity between the medial prefrontal cortex and posterior regions supporting episodic and semantic representations (Demblon et al., 2016).

## Autobiographical thinking and psychopathology

Many psychological disorders—including depression, anxiety, schizophrenia, substance abuse, and personality disorders—are associated with deficits and/or biases in autobiographical memory and future thinking (for reviews, see Brunette & Schacter, 2021; Hallford et al., 2018; Williams et al., 2007). Understanding the mechanisms underlying these alterations and designing interventions to address them became one of Martial’s most important interests in the latter part of his career.

Stéphane Raffard and I contributed to this endeavour by looking specifically at disturbances of autobiographical thinking in schizophrenia. To my knowledge, we were the first to provide formal evidence that individuals with schizophrenia not only have difficulties in remembering specific events from their personal past but are even more impaired at imagining specific events that might happen in their personal future (D’Argembeau et al., 2008). These difficulties in remembering the past and imagining the future may in part be due to deficits in the process of scene construction (i.e., the ability to mentally generate and maintain a complex and coherent scene). Indeed, when attempting to construct mental representations of scenes, individuals with schizophrenia report fewer sensory descriptions and spatial references than healthy individuals, and their descriptions lack spatial coherence and are more fragmented (Raffard, D’Argembeau, Bayard, et al., 2010).

Considering the role of autobiographical memories and future thoughts in the development and maintenance of the self, these deficits in autobiographical thinking might in part explain the alterations in the sense of continuity of self that are observed in schizophrenia (Mishara et al., 2014). To examine this possibility, we conducted a series of studies to investigate the characteristics of self-defining memories and future thoughts in schizophrenia. We found that patients recall as many specific self-defining memories as healthy individuals, but their narratives are less elaborate and include fewer connections with the self (Raffard, D’Argembeau, Lardi, et al., 2010; Raffard et al., 2009). In the same vein, they have difficulty thinking about the broader meaning and implications of important future events (Raffard et al., 2016). Furthermore, the peak of the temporal distribution of self-defining memories (the so-called reminiscence bump; Rubin et al., 1998) occurs at an earlier age in patients with schizophrenia, which might reflect alterations in identity development (Raffard, D’Argembeau, Lardi, et al., 2010). Taken together, these findings suggest that patients with schizophrenia have difficulty organizing and extracting meaning from their experiences to create coherent self-narratives.

## An integrative framework of the cognitive architecture of autobiographical thinking

The research program that I initiated with Martial more than 15 years ago (and that I have pursued with my team to this day) has recently led me to propose a theoretical framework to account for the cognitive architecture of autobiographical thinking (D’Argembeau, 2020). This integrative framework is very much inspired by Martin Conway’s view on the organization of autobiographical memory (Conway, 2005; Conway & Pleydell-Pearce, 2000) and extends its scope to future thinking (Conway et al., 2019).

The basis of this proposal is that autobiographical thinking relies on two interacting systems, namely event simulation and autobiographical knowledge. The event simulation system allows one to mentally simulate specific episodes based on details from prior experiences (drawn from episodic memory; Schacter & Addis, 2007) and semantic knowledge (notably, event schema; Irish & Piguet, 2013). Such simulations rely on mental imagery and modality-specific systems for perception, action, emotion, and introspection (Barsalou, 2008), their function being to provide a representation of the experiential content of events from an egocentric perspective—they depict what it would be like to experience the simulated event. Remembering past events and imagining future events both involve simulation processes (Addis, 2020), but as such, event simulations are atemporal in nature, in that they lack a broader temporal context that situates events in the past, present, or future. The temporal context of event simulations is provided by autobiographical knowledge, which forms a cognitive representational system—a personal timeline—onto which simulated events can be mapped. Autobiographical knowledge involves conceptual/propositional representations, which organize information about the content and structure of one’s past, present, and future life in hierarchical layers referring to different temporal scopes (from lifetime periods that encompass years to events occurring on specific days). According to this view, the subjective temporality of memories and future thoughts does not lie in event simulations per se but in the synergy between event simulations and autobiographical knowledge (for more detail, see D’Argembeau, 2020).

The nature and level of specificity of autobiographical thinking depends on which system is active at a given moment. Autobiographical knowledge about the past and future (including knowledge about the occurrence of specific events) can be accessed without necessarily representing the experiential content of events, resulting in abstract forms of autobiographical thinking (e.g., quickly reviewing the main chapters that constitute one’s life; Thomsen, 2009). The subjective experience of “revisiting” the past or “pre-experiencing” the future occurs only when event simulations are constructed and integrated with higher-order autobiographical knowledge, resulting in the feeling of mental time travel (D’Argembeau & Van der Linden, 2012; Ernst & D’Argembeau, 2017). The diversity of autobiographical thoughts we experience in daily life—concrete or abstract, imagery-based or language-based, near or far, past or future—is generated by the constant dance of the two systems.

## Conclusion

Memories of significant events are like pieces of a puzzle that create parts of our identity. The moments I remember spending with Martial certainly played such a role in shaping my identity as a researcher and I am grateful to him for that. The research we initiated together has contributed to a better understanding of how the weaving of our memories defines who we are now and shapes who we aspire to be in the future. Shedding further light on the nature of autobiographical thought processes might ultimately help people restore meaning and purpose in life, a much needed endeavour in these difficult times (de Jong et al., 2020).
